# THE SOUTHEAST FLORIDA VETERANS’ COMMUNITY PERSPECTIVES ON THE MEANING OF WELL-BEING: A PHENOMENOLOGICAL INQUIRY

**DOI:** 10.1093/geroni/igad104.3666

**Published:** 2023-12-21

**Authors:** Cheryl Birmingham

**Affiliations:** Florida Atlantic University, Fort Lauderdale, Florida, United States

## Abstract

The Veterans are a unique community shaped by past military experiences that may have positive and negative effects on their well-being as soldiers. With nearly half of the 18.4 million Veterans in the United States living beyond 61 years the physical and mental health conditions may desolate the Veterans’ well-being. Promoting Veteran well-being has been studied extensively but scant on how they define it. It is critical to understand how Veterans define well-being to inform future program development addressing mental health needs. The purpose of this study is to discover the meaning and the live experience of well-being among Veterans. A qualitative hermeneutic interpretative phenomenological approach was used to understand the meaning of Veterans’ well-being. It allowed the phenomena to surface while exposing the essence of and the experience. Purposive sampling was used to recruit participants, with semi-structured interviews via ZOOM and observations at Veteran Service Organizations (VSOs). Fourteen Veterans participated in this study, 8 males and 6 females with majority greater than 61 years. 64% were White, 21% Hispanic and 15% African American. With the use of Max van Manen’s analysis strategy, 3 major themes emerged: (1) A Connection to a Military Sisterhood/Brotherhood; (2) The Lingering Effects of Military Service and (3) Healthy Companionship/Relationships advocating for Veterans’ well-being. The lingering effects persisted and challenged the Veterans’ emotional well-being; however, they were able express their military camaraderie continued in Veteran life. Programs based on social relationships that allow connections with Veterans and/or their significant others need to be established.

